# Serum and Glucocorticoid Regulated Kinase 1 in Sodium Homeostasis

**DOI:** 10.3390/ijms17081307

**Published:** 2016-08-10

**Authors:** Yiyun Lou, Fan Zhang, Yuqin Luo, Liya Wang, Shisi Huang, Fan Jin

**Affiliations:** 1 Department of Reproductive Endocrinology, Women’s Hospital, School of Medicine, Zhejiang University, Hangzhou 310006, Zhejiang, China; louyiyun@zju.edu.cn (Y.L.); zhangfanchina@zju.edu.cn (F.Z.); szyck@zju.edu.cn (Y.Lu.); wangliya@zju.edu.cn (L.W.); huangshisi@zju.edu.cn (S.H.); 2 Department of Gynaecology, Hangzhou Hospital of Traditional Chinese Medicine, Hangzhou 310007, Zhejiang, China; 3 Key Laboratory of Reproductive Genetics, National Ministry of Education (Zhejiang University), Women’s Reproductive Healthy Laboratory of Zhejiang Province, Hangzhou 310058, Zhejiang, China

**Keywords:** serum and glucocorticoid regulated kinase 1 (SGK1), epithelial sodium channels, voltage-gated sodium channels, hypertension, edema, heart disease, embryo implantation

## Abstract

The ubiquitously expressed serum and glucocorticoid regulated kinase 1 (SGK1) is tightly regulated by osmotic and hormonal signals, including glucocorticoids and mineralocorticoids. Recently, SGK1 has been implicated as a signal hub for the regulation of sodium transport. SGK1 modulates the activities of multiple ion channels and carriers, such as epithelial sodium channel (ENaC), voltage-gated sodium channel (Nav1.5), sodium hydrogen exchangers 1 and 3 (NHE1 and NHE3), sodium-chloride symporter (NCC), and sodium-potassium-chloride cotransporter 2 (NKCC2); as well as the sodium-potassium adenosine triphosphatase (Na^+^/K^+^-ATPase) and type A natriuretic peptide receptor (NPR-A). Accordingly, SGK1 is implicated in the physiology and pathophysiology of Na^+^ homeostasis. Here, we focus particularly on recent findings of SGK1’s involvement in Na^+^ transport in renal sodium reabsorption, hormone-stimulated salt appetite and fluid balance and discuss the abnormal SGK1-mediated Na^+^ reabsorption in hypertension, heart disease, edema with diabetes, and embryo implantation failure.

## 1. Introduction

Serum and glucocorticoid regulated kinase 1 (SGK1) was originally isolated in a differential screen searching for glucocorticoid-inducible transcripts in Con8.hd6 rat mammary tumor cells [1,2]. Within 30 min, *SGK1* transcript levels were altered strongly upon cell volume change, independent of de novo protein synthesis [3]. *SGK1* is highly conserved throughout eukaryotic evolution [4], being identified in the genomes of various species [5,6,7,8,9,10,11,12,13,14,15]. Human *SGK1* is ubiquitously expressed throughout the whole body (Table 1).

As a serine-threonine protein kinase, SGK1 belongs to the protein kinase A/protein kinase G/protein kinase C (AGC) family, and is expressed at low levels under physiological conditions [47,48,49]. Both its expression levels and activities are regulated by hormonal and non-hormonal factors [50], including glucocorticoids [51,52], mineralocorticoids [16,42], androgen [53,54,55,56], gonadotropin-releasing hormone (GnRH) [57], excessive extracellular glucose concentrations [58,59], memory consolidation and reconsolidation [60], hypertonic and hypotonic stimuli [61], chronic stress [52], and peroxisome proliferator-activated receptor γ (PPARγ) [62]. SGK1 is also induced by lipopolysaccharides [63], tumor necrosis factor (TNF)-α [63,64], angiotensin [65], resistin [66], granulocyte-macrophage colony-stimulating factor (GM-CSF) [67], fibroblast growth factor-23 (FGF23) [68], as well as miR-27a [24], miR-424 [69], miR-155 [70] and miR-133b [71]. Under stimulation by transforming growth factor (TGF)-β [72,73] and insulin [74], SGK1 is phosphorylated via signaling pathways involving phosphatidylinositol 3-kinase (PI3K), 3-phosphoinositide-dependent kinases (PDK1) [74] and mammalian target of rapamycin complex 2 (mTORC2) [75,76]. By contrast, interleukin-6 (IL-6) induces *SGK1* transcription mainly through the janus kinase/signal transducer and activator of transcription (JAK/STAT) cascade [77]. These genomic and non-genomic activations of SGK1 contribute to the regulation of multiple epithelial ion channels, several ion carriers, and many other molecules [78].

The first demonstrated physiologically relevant function of SGK1 was its regulation of ENaC-mediated Na^+^ transport [79]. The present review attempts to delineate the current knowledge on the physiological and pathophysiological significance regarding SGK1 in the regulation of Na^+^ homeostasis.

## 2. Serum and Glucocorticoid Regulated Kinase 1 (SGK1)-Dependent Regulation of Na^+^ Channels and Transporters

### 2.1. Epithelial Sodium Channel (ENaC)

Over the past 20 years, SGK1 has emerged as a key modulator of ENaC in the aldosterone-sensitive distal nephron (ASDN) [80], hepatocytes [81], lung [82], corneal layers [22], and brain [83]. SGK1 increases the amiloride-sensitive Na^+^ current significantly in *Xenopus laevis* oocytes [81,84], mouse collecting duct cells (mpkCCD_cl4_) [85], mammalian M1-CCD cells [86], amphibian A6 cell line [87,88], COS7 cells [89], H441 human airway epithelial cells [90,91], and colonic HT-29/B6 cells [92].

Upon stimulations of hormonal and non-hormonal signals, SGK1 regulates Na^+^ transport in various cells by altering ENaC expression [93,94], enhancing this channel’s activity and open probability (*P*_o_) [95], facilitating ENaC channel trafficking, and attenuating its degradation and recycling [96].

Several mechanisms have been proposed for the SGK1-dependent regulation of ENaC [82,97]. The best understood explanation argues that aldosterone-induced SGK1 increases ENaC activity indirectly by reducing ubiquitination of ENaC via phosphorylation and inhibition of the E3 ubiquitin ligase neuronal precursor cell expressed developmentally down-regulated 4-2 (Nedd4-2) [93], which results in increased Na^+^ transport in *Xenopus laevis* oocyte. SGK1 phosphorylates specific residues of Nedd4-2, resulting in the recruitment of the 14-3-3 protein, which inhibits the interaction between Nedd4-2 and ENaC. This inhibition is dependent on SGK1-catalyzed phosphorylation of Nedd4-2 [98,99]. Consistent with this view, GSK650394, an SGK1 inhibitor, suppresses the dexamethasone-induced phosphorylation of Nedd4-2, and reduces the surface abundance of α subunit of ENaC in airway epithelial cells [91]. Therefore, SGK1 phosphorylates the negative regulator Nedd4-2 and recruits 14-3-3, thereby preventing the ubiquitination and subsequent internalization of ENaC, and inhibiting the removal of the channel. This results in accumulation of ENaC at the cell surface and increased Na^+^ reabsorption as reviewed in [100,101,102,103,104,105].

In the above model, ENaC, possibly with cholesterol, recruits proteins to form the ENaC-regulatory complex (ERC) for its own regulation [106,107]. In this respect, Soundararajan et al. [108] have identified an approximately 1.0–1.2 MDa ENaC-regulatory-complex (ERC) containing ENaC and certain key regulatory factors, including aldosterone-regulated SGK1, Nedd4-2, v-raf-1 murine leukemia viral oncogene homolog 1 (c-Raf), glucocorticoid-induced leucine zipper (GILZ1), and the connector enhancer of kinase suppressor of Ras isoform 3 (CNK3), at the plasma membrane in mpkCCD_c14_ cells [107,108]. GILZ1 physically interacts with SGK1 to alter its subcellular localization and selectively recruits it into the ERC [106]. Contrastingly, CNK3 reinforces the interactions within this complex, providing a platform to assemble the multiprotein ERC to trigger ENaC activation [108,109,110].

Moreover, IκB kinase-β (IKKβ) was shown recently to enhance ENaC surface expression by phosphorylating Nedd4-2 on the same site phosphorylated by SGK1 [111,112]. Stimulated by serum in MDA231 cells derived from human breast cancer [113] or using morpholino oligonuleotides against SGK1 in *Xenopus laevis* oocyte [114], SGK1 was demonstrated to function upstream of IKKβ; therefore, SGK1 could modulate the activities of Nedd4-2 in concert with IKKβ, contributing to the enhanced accumulation of ENaC channel at the apical membrane [98,111].

While the SGK1/Nedd4-2 pathway could lead to enhanced ENaC function [101,111], other studies point to alternative pathways for SGK1 to regulate ENaC activity, independently of Nedd4-2 [110,115]. In this regard, recombinant SGK1 has been shown to directly phosphorylate residue serine (Ser)-621 of the SGK1 consensus motif in the C terminus tail of α-ENaC in *Xenopus laevis* oocytes, contributing to the activation of ENaC channels that are already present in the plasma membrane [116].

Recent evidence has demonstrated that SGK1 also has a role in aldosterone-stimulated ENaC trafficking in mCCD cells. This mode of channel regulation involves the Rab GAP (GTPase activating protein) AS160, Akt/PKB substrate of 160 kDa, which stabilizes ENaC in a regulated intracellular compartment [117]. Upon SGK1 phosphorylation, AS160 promotes ENaC trafficking to the apical membrane by relieving stabilization of ENaC in the intracellular compartment, thus augmenting Na^+^ absorption [117]. In addition, FLAG-tagged SGK1 has been implicated in the regulation of ENaC in HEK293 cells by phosphorylating and thus inhibiting with no lysine kinase 4 (WNK4) [118,119], a serine/threonine kinase that inhibits ENaC activity [120]. SGK1 further regulates ENaC indirectly by phosphorylating inducible nitric oxide synthase (iNOS) [121]. Nitric oxide (NO) inhibits ENaC by reducing its *P*_o_ without altering the apparent channel density or Na^+^ current [121,122]. Upon the stimulation of aldosterone, SGK1 phosphorylates mouse iNOS and consequently decreases NO produced by iNOS to increase Na^+^ transport in the mouse alveolar type II (ATII) epithelial cells [121].

SGK1 is proposed to up-regulate [123] or de-repress [124] the components of the Na^+^ transport machinery per se, primarily α-ENaC. Evidence from *Sgk1* knockout mice and mouse inner medullary collecting duct cell (mIMCD3) indicated that aldosterone-induced SGK1 is involved in an epigenetic pathway regulating the transcription of *SCCH1A* (gene encoding α-ENaC) by diminishing hypermethylation of histone protein H3 at lysine 79 (H3K79) in the vicinity of the *SCCH1A* promoter [124]. SGK1 phosphorylates DNA-binding protein ALL1 fused gene from chromosome 9 (AF9), and thus promotes methyltransferase Disruptor of telomeric silencing 1 (Dot1a) to dissociate from the *SCCH1A* promoter, leading to inhibition of histone H3K79 methylation at the promoter and subsequently relief of repression [124,125,126]. Interestingly ALL-1 partner at 17q21 (AF17), a competitor of AF9 for binding Dot1a, relieves Dot1a-AF9 repression as well as increasing *SGK1* expression to enhance SGK1-mediated AF9 phosphorylation, resulting in augmented ENaC-mediated Na^+^ transport [127,128,129,130,131].

**Figure 1:**
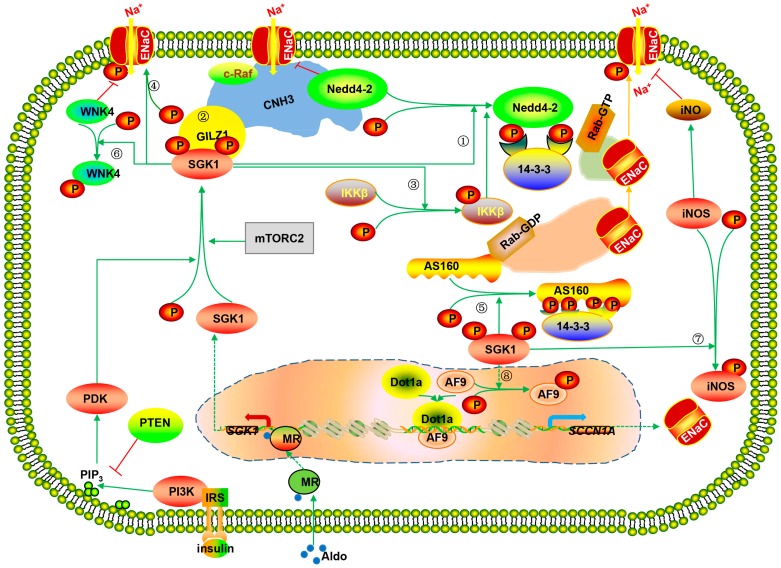
Serum and Glucocorticoid Regulated Kinase1 (SGK1)-dependent regulation of ENaC channel. ① SGK1 phosphorylates the negative regulator Nedd4-2 and recruits 14-3-3 protein to reduce the ubiquitylation and degradation of ENaC; ② SGK1 interacts with GILZ1, CNK3, c-Raf, ENaC and Nedd4-2 to form the ENaC-regulatory complex (ERC) for stimulating ENaC function; ③ SGK1 phosphorylates IKKβ to reverse the Nedd4-2-mediated inhibition of ENaC; ④ SGK1 directly phosphorylates α subunit of ENaC; ⑤ SGK1 phosphorylates AS160 to promote ENaC trafficking to the apical cell membrane; ⑥ SGK1 activates ENaC via phosphorylating WNK4; ⑦ SGK1 enhances the open probability of ENaC channel by decreasing inhibitory NO through phosphorylating iNOs; and ⑧ SGK1 is involved in an epigenetic pathway regulating *SCCH1A* (gene encoded α-ENaC) transcription by phosphorylating AF9 and promoting Dot1a to dissociate from SCCH1A promoter, diminishing the hypermethylation of histone H3K79 methylation at the promoter of SCCH1A. The translocations of moleculars are marked in dashed arrows. The red arrows with flat head mean inhibitory modification. P, phosphate; PTEN, phosphatase and tensin homolog; IRS, insulin receptor substrate; MR, mineralocorticoid receptor; Aldo, aldosterone.

Taken together, SGK1 regulates ENaC activity through Nedd4-2-dependent and Nedd4-2-independent mechanisms [101,111] (Figure 1). These mechanisms are not mutually exclusive. Upon the stimulation of hormonal (e.g., aldosterone, dexamethasone) or non-hormonal (e.g., serum) signals, the activation of SGK1 attenuates the degradation of ENaC to increase the surface abundance of this Na^+^ channel at the apical membrane [91,93,106,113,114,118,119], relieves the stabilization of ENaC in a regulated intracellular compartment [117], and facilitates ENaC activities by direct phosphorylation [116,117] in various cell lines. Moreover, aldosterone-induced SGK1 has long-term effect on the transcriptional expression of ENaC in an epigenetic pathway both in vivo and in vitro [124]. The discrepancies among the various mechanisms could be ascribed to the characteristics of different stimuli and the timing of SGK1’s action.

### 2.2. Voltage-Gated Na^+^ Channel Nav1.5 (SCN5A)

The voltage-gated sodium channel Nav1.5 (encoded by the *SCN5A* gene), is the major Na^+^ influx channel for the cardiac action potential initiation of cardiac action [132]. As shown in *Xenopus laevis* oocytes, SGK1 up-regulates cardiac Nav1.5 [97,133]. Through phosphorylation and inactivation of Nedd4-2, SGK1 attenuates the inhibition on Nav1.5 by Nedd4-2, and alters channel trafficking, resulting in an increase in available Nav1.5 channels at the cell surface [97,133,134,135]. Conversely, inhibition of SGK1 in the dominant-negative *Sgk1* mice blocked the biochemical changes in Nav1.5 [134]. In addition, peptide mapping identified three putative phosphorylation sites for SGK1 within the Nav1.5 sequence [132,134]. The mutation of serine to alanine in the SGK consensus sequences of Nav1.5 resulted in a reversal of the gating properties of the channel [97,133]. More recently, Bezzerizes et al. [132] observed that an alanine mutant abolished the increase in Na^+^ current from SGK1 activation. Thus SGK1 might modify the gating kinetics of Nav1.5 channels by direct phosphorylation of the channel protein [97].

### 2.3. Sodium Hydrogen Exchanger (NHE1 and NHE3)

NHE3 participates in Na^+^ reabsorption and H^+^ secretion in a variety of epithelia and is involved in the modulation of cytosolic pH in various epithelial and non-epithelial cells [48,136,137,138]. In cultured epithelial cells, SGK1 enhances NHE3 activity acutely [139,140]. SGK1 specifically phosphorylates NHE3 at Ser-663 in response to dexamethasone; therefore, mutation of Ser-663 abolished the stimulatory effect of dexamethasone on NHE3 transport activity [139]. This up-regulation requires the additional presence of the NHE regulatory protein 2 (NHERF2) [141], which tethers NHE3 and SGK1 to aid the phosphorylation of NHE3. Comparing short-term regulation of NHE3 by dexamethasone in *Sgk1^flox/flox^*:*Villin-Cre* mice and *Nherf2^−/−^* mice, He et al. [142] showed that SGK1 plays a major role in acute regulation of NHE3 in vivo in the intestine.

Critically, SGK1 participates in the up-regulation of NHE1 by glucocorticoids in HL-1 cardiomyocytes in vivo [143,144]. Activation of NHE1 could induce cardiac hypertrophy and unbalanced cardiomyocyte pH, which may lead to myocardial remodeling and ischemic cardiac diseases [145,146,147]. SGK1 presumably phosphorylates NHE1 at Ser-703, promoting 14-3-3 binding and stimulating NHE1 activity by decreasing dephosphorylation and by stabilizing an active conformation of the exchanger [50,143]. Stimulated by dexamethasone, SGK1 would participate in the development of heart failure and other cardiac pathophysiology by activating cardiac NHE1 [143].

### 2.4. Sodium-Chloride Symporter (NCC)

The Na^+^-Cl^−^ cotransporter, sodium-chloride symporter (NCC), is expressed in the apical plasma membrane of epithelial cells in the distal convoluted tubule (DCT) [148,149]. NCC reabsorption accounts for only 5%–10% of filtered Na^+^; however, is critical to the fine-tuning of renal sodium excretion in response to various hormonal and non-hormonal stimuli [149,150]. NCC can be regulated by changes in expression, trafficking and phosphorylation [151].

The total *Sgk1* knockout mice generated by Fejes-Tóth et al. [80] exhibited a salt-wasting phenotype under a low salt diet, had reduced ENaC expression and decreased expression of NCC [84]. This phenotype was similar to that of kidney-specific *Sgk1* knockout mice [80]. Furthermore, on a low-NaCl diet, NCC abundance in the DCT of normal mice increased as did phosphorylation of NCC at Thr-53, Thr-58, and Ser-71 [148]. This response, however, is attenuated in mice lacking *Sgk1* (*Sgk1^−/−^*), suggesting that Sgk1 somehow affects NCC phosphorylation [148].

SGK1 is thought to modulate NCC activity by inhibiting WNK4 [120,149,152]. WNK4 negatively regulates the surface abundance of NCC by promoting lysosomal degradation [153]. Moreover, WNK4 has been demonstrated to reduce NCC abundance at the plasma membrane, resulting in the inactivation of NCC [154]. Constitutively active SGK1_S422D_ phosphorylates WNK4 at Ser-1169 [118] and Ser-1196 [155], relieving the inhibitory effect of WNK4 on NCC’s activity [149,151].

In addition, aldosterone acutely stimulated Na^+^ reabsorption by NCC in the DCT, and this effect appeared to be dependent upon the presence of SGK1 and Nedd4-2 [156,157]. Accordingly, SGK1 has been proposed to be involved in the regulation of NCC by Nedd4-2 [158]. Similar to ENaC, Nedd4-2 is co-located with NCC and stimulates NCC ubiquitination at the apical plasma membrane. Phosphorylation of Nedd4-2 at Ser-328 and Ser-222 by SGK1 abrogates Nedd4-2-mediated inhibition of NCC [156]. Roy et al. proposed that SGK1 and Nedd4-2 cannot alter the phosphorylation status of NCC in *WNK1* KO HEK-293T cells, representing another model of the effects of WNK1 deletion on Nedd4-2/SGK1 regulation of NCC [150].

### 2.5. Na^+^-K^+^-2Cl^−^ Cotransporter (NKCC2)

SGK1 is not only involved in the regulation of ENaC, but also influences other renal tubular Na^+^ transport systems [159]. The Na^+^-K^+^-2Cl^−^ cotransporter (NKCC2 or BSC-1) is one of the candidate downstream effectors. NKCC2 plays a critical role in Na^+^ reabsorption and urinary K^+^ excretion across the luminal membrane of the thick ascending limb (TAL) [160]. NKCC2-mediated Na^+^ flux was stimulated 6-fold by the co-expression of SGK1 in *Xenopus laevis* oocytes [161]. Stimulated by the increased extracellular glucose concentrations, the enhanced expression of *SGK1* may contribute to the abnormal Na^+^ transport in diabetic nephropathy by regulating NKCC2 [160].

### 2.6. Sodium/Potassium-Adenosine Triphosphatase (Na^+^/K^+^-ATPase)

SGK1 has also been implicated in the regulation of Na^+^/K^+^-ATPase activity, the transporter responsible for basolateral Na^+^ efflux [162]. SGK1 co-localizes with the Na^+^/K^+^-ATPase in renal epithelial cells [162]. In *Xenopus laevis* oocytes, SGK1 increased the activity of both endogenous and exogenous Na^+^/K^+^-ATPase [48,97,163,164,165,166]. In A6 cells derived from the *Xenopus laevis* distal tubule, SGK1 expression increases Na^+^/K^+^-ATPase activity, independent of changes in abundance at the plasma membrane or protein expression [162]. Constitutively active mutant of SGK1 (SGK1_S425D_) stimulates existing Na^+^ pumps in the basolateral plasma membrane for the Na^+^ exiting [162], which would maintain the chemical driving force for Na^+^ entry through ENaC [162]. In addition, the stimulatory effect of SGK1 on Na^+^/K^+^-ATPase is mimicked by the isoforms SGK2 and SGK3 in *Xenopus laevis* oocytes [167].

### 2.7. Type A Natriuretic Peptide Receptor (NPR-A)

The human isoform of *SGK1* has been identified as a cell volume-regulated gene that is modulated transcriptionally by cell swelling and shrinkage [3,168,169]. Accordingly, SGK1 has been shown to be involved in the extracellular tonicity-dependent stimulation of the NPR-A gene promoter in rat inner medullary collecting duct (IMCD) cells via the p38 mitogen-activated protein kinase (MAPK)-dependent pathway [170]. Beyond that, hypertonicity induces the expression of tonicity-responsive enhancer binding protein/nuclear factor of activated T cells 5 (TonEBP/NFAT5), which accounts for the osmosensitivity of the *SGK1* gene promoter [61]. In turn, SGK1 does indeed serve as a key mediator in the osmotic induction of *NPR-A* gene expression [61].

Taken together, SGK1 acts as a key intracellular signal that regulates the activities of ENaC, Nav1.5, NHE1 and NHE3, NCC, NKCC2, Na^+^/K^+^-ATPase, and NPR-A, thus contributing to Na^+^ homeostasis (Table 2).

## 3. Physiological Role of SGK1 in Na^+^ Transport

### 3.1. SGK1-Dependent Renal Na^+^ Reabsorption

The kidneys play a pivotal role in the maintenance of Na^+^ homeostasis [62,173]. Urinary Na^+^ reabsorption is regulated tightly to maintain a constant extracellular volume as limiting extrarenal Na^+^ loss during dietary Na^+^ restriction [47]. The final adjustment to renal Na^+^ balance is achieved in the ASDN: i.e., the distal convoluted tubule (DCT), the connecting tubule (CNT), the cortical collecting duct (CCD) and the medullary collecting duct (MCD) [120,163]. Aldosterone and vasopressin play major roles in regulating Na^+^ flux in epithelial tissues in these segments [85,174,175]. This effect is accomplished by the coordinated action of Na^+^ entry into the epithelial cells via ENaC channel on the apical membrane, as well as Na^+^ exit through the Na^+^/K^+^-ATPase pump on the basolateral plasma membrane [47,176].

As illustrated above, SGK1 regulates ENaC [62,85,173] in the apical membrane and the Na^+^/K^+^-ATPase in the basolateral membrane, thereby coordinating Na^+^ transport at both sides of epithelial cells [177]. In early distal tubules, the chlorothiazide-sensitive NCC mediates Na^+^ uptake [178,179]. SGK1 phosphorylates Nedd4-2 and WNK4, blocking their inhibitory effects on NCC [180]. In addition to stimulating Na^+^ uptake in the ASDN, SGK1 participates in Na^+^ transport in other renal segments. In rats and mice on a standard NaCl diet, expression of *Sgk1* mRNA was detected in the glomeruli, proximal tubules [181], ASDN, and particularly strongly, in the IMCD [32,182]. SGK1 protein is localized to the TAL and ASDN [163], whereas very low protein expression was detected under basal conditions in the glomeruli, proximal tubule or MCD, including the papilla in rat kidneys [181,182]. Therefore, apart from ENaC, SGK1 increases Na^+^ reabsorption via various transporters: NHE3 in the proximal tubule (PT) [136,183,184]; NKCC2 in the loop of Henle of TAL; as well as the Na^+^ pump in different nephron segments [180].

The central role of SGK1 in the hormonal control of Na^+^ handling is further illustrated by the observations in mice lacking *Sgk1* [80,185,186,187]. Under a normal-salt diet, the phenotype of the *Sgk1^−/−^* mouse was virtually identical to that of its wildtype littermates (*Sgk1^+/+^*) [80,178,186,188]. These *Sgk1^−/−^* mice showed no obvious defect in water and Na^+^ excretion, and maintained normal apical membrane staining for α-ENaC in the connecting tubule, except for higher circulating aldosterone levels, suggesting extracellular volume depletion [188,189]. However, when exposed to an NaCl-deficient diet, the *Sgk1^−/−^* mice presented a dramatic urinary salt wasting phenotype: weight loss caused by increased urine production, decreased systolic and diastolic blood pressure, increased urinary Na^+^ and K^+^ excretion with unchanging plasma Na^+^ and K^+^ levels, and higher plasma aldosterone [80,188]. Wulff et al. [188] reported a weaker amiloride-sensitive transepithelial transport potential difference in isolated collecting ducts (CD) of *Sgk1^−/−^* mice compared with *Sgk1^+/+^* mice. In contrast, Fejes-Tóth et al. [80] reported increased amiloride-sensitive Na^+^ currents with decreased γ-ENaC cleavage, as well as diminished NCC protein expression, in isolated collecting ducts of *Sgk1^−/−^* mice compared with the wildtype mice. Recently, Faresse et al. [186] generated doxycycline-inducible nephron tubule-specific *Sgk1* knockout mice (*Sgk1^Pax8/LC1^*), in which *Sgk1* expression could be repressed within the kidney by treatment with doxycycline in the drinking water. The *Sgk1^Pax8/LC1^* mice also exhibit a large defect in Na^+^ conservation when placed on a low-Na^+^ diet [186]. *Sgk1^Pax8/LC1^* mice have a decreased expression of the βγ-ENaC protein, without any change in γ-ENaC cleavage and *α-ENaC* mRNA expression [186]. Moreover, a significant reduction of NCC protein and no difference in mRNA levels has been observed in *Sgk1^Pax8/LC1^* mice, along with decreased phosphorylation of Nedd4-2 on Ser-222 and Ser-328 by Sgk1. This finding suggests a potential SGK1-dependent regulation of NCC in renal Na^+^ reabsorption [186].

### 3.2. SGK1-Dependent Renal Na^+^ Excretion

**Figure 2:**
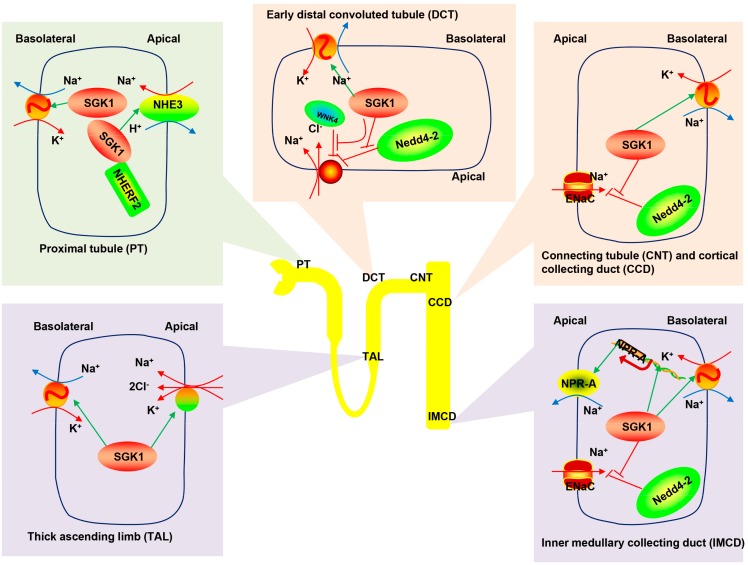
SGK1-dependent Na^+^ reabsorption and excretion in the mammalian kidney tubule. SGK1 boosts Na^+^ reabsorption via multiple transporters in different renal segments: NHE3 in the PT, NKCC2 in the loop of Henle TAL, sodium-chloride symporter (NCC) in the early distal convoluted tubule (DCT), ENaC primarily in the connecting tubule (CNT) and cortical collecting duct (CCD), as well as Na^+^-K^+^-ATPase pump throughout different nephron segments. SGK1 regulates Na^+^ excretion in the medullary collecting duct (MCD) by activating NPR-A. SGK1, serum and glucocorticoid regulated kinase 1; NHE3, sodium hydrogen exchanger 3; NHERF2, NHE regulatory protein 2; WNK4, with no lysine kinase 4; Nedd4-2, neuronal precursor cell expressed developmentally down-regulated 4-2; ENaC, epithelial sodium channel; NPR-A, type A natriuretic peptide receptor.

Since its discovery in 1993, SGK1 was first identified in the response to cell volume alterations in a human hepatoma cell line [168,169]. Cell shrinkage leads to a rapid induction of SGK1 transcription in different cell lines [61,92,168,190,191,192,193,194,195,196]. Hypertonicity in the early phase leads to an acute increase in urinary sodium excretion [61,170]. In rat IMCD cells, SGK1 transcription is modulated by tonicity-responsive enhancer (TonE) binding protein (TonEBP/NFAT5) [61], which in turn activates NPR-A, resulting in sodium excretion [61,170]. As demonstrated in rat and mouse assays, increased extracellular osmolality does indeed increase *Sgk1* and *Npr-A* gene expressions concomitantly in the MCD. Furthermore, Chen et al. [61] reported that natriuretic peptide receptor 1 (*Npr1*) gene knockout mice (*Npr1^−/−^*) failed to elicit changes in urinary Na^+^ excretion when challenged with dehydration, despite elevated urinary osmolality and *Sgk1* expression in the renal medulla. Collectively, these findings defined the contribution of the osmosensitive gene *SGK1* to medullary sodium excretion (Figure 2), where it promotes the physiological response of the kidney to dehydration [61].

### 3.3. Aldosterone-Induced Salt Appetite

In addition to regulating renal Na^+^ transport, SGK1 is thought to be involved in the regulation of aldosterone-induced salt adaptation and salt appetite [47,50,197,198,199]. When treated with deoxycorticosterone-acetate (DOCA)/1% NaCl, *Sgk1^+/+^* mice exhibited a pronounced increase in Na^+^ intake and proteinuria compared with *Sgk1^−/−^* mice [197]. The observation of pregnant mice further confirmed the role of SGK1 in the enhanced salt appetite as the preference for saline water was significantly stronger in *Sgk1^+/+^* mice than in *Sgk1^−/−^* mice [200]. Therefore, SGK1 were expected to participate in the increased salt uptake during pregnancy, contributing to the increase extracellular fluid volume, which favors hypertension of pregnancy [200].

Although the underlying mechanism remains to be exploited, Vallon et al. [197] have proposed that SGK1 might contribute to the stimulation of salt appetite in response to mineralocorticoid excess by upregulating the activity of Na^+^/K^+^-ATPase in the amygdala, an area implicated in the modulation of salt appetite. Fu et al. [199] have assumed that aldosterone activates SGK1, Nedd4-2 and ENaC in both kidney and brain. They suggested that SGK1 and ENaC were involved in aldosterone-induced salt appetite, as ENaC also mediates the gustatory salt sensing.

Thus, SGK1 appears to play a dual role in hormone-regulated Na^+^ homeostasis, attenuating urinary salt output by regulating ENaC-mediated renal Na^+^ reabsorption on the one side, and increasing salt intake through stimulating salt appetite on the other [197,200].

Notably, the dual effects converge to expand the extracellular volume, which is supposed to favor salt-sensitive hypertension [201,202,203,204,205,206].

### 3.4. SGK1-Dependent Intestinal Sodium Absorption

Under basal conditions, *SGK1* is expressed robustly in the distal colon, ileum and jejunum, which are beyond the aldosterone-responsive segments, suggesting a constitutive role in absorptive epithelia [207]. Consistently, ENaC, which is phosphorylated regulated by SGK1, plays a pivotal role in minimizing intestinal water and sodium losses in the distal colon [208]. Therefore, Dames et al. [208] showed that decreased *SGK1* expression due to the suppression of interleukin-13 (IL-13) impaired epithelial sodium absorption via ENaC.

Aldosterone-induced intestinal Na^+^ absorption is also mediated by apical Na^+^-H^+^-exchangers (NHE2/3) and basolateral Na^+^/K^+^-ATPase [209]. SGK1 has been proposed to be part of this cascade [140]. Using human colonic Caco-2 and opossum kidney cells, Wang et al. [140] observed a biphasic activation of NHE3, which is responsible for the electrogenic Na^+^ absorption in the intestinal epithelium. Furthermore, Musch et al. [210] demonstrated a potential role for SGK1 in the two phases of aldosterone-induced intestinal Na^+^ absorption. The initial phase involves enhanced insertion of the α-subunit of Na^+^/K^+^-ATPase through a PI3K-SGK1-dependent pathway and subsequently increased levels of apical membrane NHE3.The later activation is mainly concerned with elevated expression and activities of total NHE3 and Na^+^/K^+^-ATPase (α-subunit), both of which are regulated by SGK1 [210].

### 3.5. SGK1-Dependent Lung Fluid Absorption

*SGK1* is expressed strongly in the lower respiratory tract. The SGK1-dependent regulation of ENaC in pulmonary epithelial cells plays a critical role in sodium/fluid homeostasis and in lung fluid clearance [90]. In this regard, several studies have reported increased *SGK1* expression in prenatal lung segments [7,211,212]. Thus, decreased *SGK1* expression could contribute to the inability to clear excessive lung fluid immediately after preterm birth [213,214].

Using H441 human airway epithelial cells, Ismail et al. [91] showed that the activation of SGK1 by dexamethasone increases the surface expression of α-, β- and γ-ENaC, while the inhibition of SGK1 suppresses the phosphorylation of Nedd4-2 and reduces the surface abundance of α-ENaC, contributing to increased membrane Na^+^ transport [91]. Furthermore, in the lipopolysaccharide (LPS)-induced acute lung injury (ALI), activation of SGK1 promotes both the total gene expression and the surface abundance of ENaC, leading to a protective effect in the case of LPS-induced ALI [74,82,94]. Therefore, SGK1 is essential to the induction and maintenance of controlled Na^+^ absorption in the respiratory system, and is involved in the hormonal management of respiratory distress and pulmonary edema, which are clinical manifestations of abnormal pulmonary Na^+^ absorption [91].

### 3.6. SGK1-Dependent Peripheral Na^+^ Transport

*SGK1* is co-expressed with ENaC in the human ocular ciliary epithelium and basal cells of corneal endothelium [74,82,94]. The activation of ENaC, NKCC2 and Na^+^/K^+^-ATPase induced by SGK1 could contribute to sodium transport in the human ocular ciliary epithelium and corneal endothelium, and further account for corneal transparency [22,23].

In the epithelium of the human middle ear, ENaC-mediated sodium transport is upregulated by dexamethasone via the glucocorticoid receptor (GR)-SGK1-Nedd4-2 pathway [25,215]. Zhong et al. [216] showed that *SGK1* is expressed in various regions of guinea pig cochlea, being associated with the regulation of endolymph homeostasis by mediating passive entry of sodium into cells. Thus SGK1 could be involved in the therapeutic activity of glucocorticoids in the treatment of Meniere’s disease, a debilitating condition that manifests endolymphatic hydrops, which might be associated with Na^+^ hypoabsorption in the vestibular lumen [25,215].

## 4. Pathological Role of SGK1 in Na^+^ Transport

### 4.1. Salt-Sensitive Hypertension

Excessive renal Na^+^ retention can increase the circulating volume which may contribute to the development of high blood pressure [189]. SGK1 participates in facilitating hormonal actions involved in stimulating salt intake and inhibiting renal sodium loss; thereby influencing the long term control of arterial blood pressure, thus contributing to the development of hypertension.

The daily salt intake seems to predispose certain individuals to develop salt-sensitive hypertension [201,217,218,219]. Sgk1 is believed to contribute to the preference for a high salt diet and be involved in hormone-induced salt adaptation [197,198]. In Dahl salt-sensitive (DS) rats, which show hypertension with a high salt diet, the renal expression of *Sgk1* is elevated greatly [201]. Furthermore, in animals receiving a high-fat diet [202] or high fructose intake [203], in addition to high salt intake, increased blood pressure is only detected in *Sgk1^+/+^* mice, but not in *Sgk1^−/−^* mice. Following high-salt intake, Sgk1-mediated up-regulation of ENaC, as well as Na^+^/K^+^-ATPase, stimulates Na^+^ transport in the cerebrospinal fluid and the brain, which would activate the renin–angiotensin system, leading to the release of ouabain-like compound (OLC) which in turn activates the renin–angiotensin system, thereby increasing blood pressure [220].

Renal salt retention is another culprit thought to be involved in the development of hypertension [96,171,200,221]. The renal re-absorption of Na^+^ is critical to whole body Na^+^ and water balance, and to the control of blood pressure [180,221]. This process, as discussed above, is accomplished partially via the mediation by SGK1. SGK1 enhances the activity of ENaC, NCC, NKCC2, and Na^+^/K^+^-ATPase, which in turn increase the Na^+^ re-absorption [189,222]. In particular, in primary aldosteronism or Liddle’s syndrome, SGK1 increases the activity of ENaC channels in response to aldosterone [223,224]. In addition, gene variants of these transporters and enzyme are also associated with increased blood pressure [189,225].

In fact, some distinct variants of the *SGK1* gene are indeed indicated in increased blood pressure [189]. Polymorphisms in intron 6 [I6CC] and exon 8 [E8CC/CT] are associated with moderately enhanced blood pressure in individuals carrying these variants [226,227,228,229,230]. These gene variants affect about 3%–5% of the Caucasian population [226,227] and 11.6% of a healthy African population [231]. In a study of 421 hypertensive Caucasian participants, Rao et al. [217] determined that two single nucleotide polymorphisms (SNPs) of SGK1 (rs2758151 and rs9402571) were associated with effects upon blood pressure and plasma renin activity (PRA) as a result of dietary salt intake. The major allele homozygotes at either rs2758151 or rs9402571 were associated with high systolic blood pressure in response to a high salt diet and decreased PRA on a low salt diet [217]. Recently, Chu et al. [232] reported that a genetic polymorphism in *SGK1* is significantly correlated with the blood pressure response to dietary sodium intervention: SNP rs9389154 was associated with systolic blood pressure (SBP), while SNPs (rs1763509 and rs9376026) were associated with diastolic blood pressure (DBP). SNP rs9376026 was significantly associated with both mean arterial pressure (MAP) and DBP, and SNP rs3813344 was significantly linked with SBP, DBP, and MAP. Accordingly, individuals with these genotypes would be prone to salt-sensitive hypertension [217,232].

Moreover, Sgk1 appears to be critical for the fetal programming of hypertension [172,233,234]. A protein-deficient diet during pregnancy leads to increased blood pressure in the offspring of *Sgk1^+/+^* mothers mice [233,234].

Taken together, dysregulation of SGK1 activity or certain specific gene variants of *SGK1* could be involved in salt-sensitive hypertension [233,234].

### 4.2. Edema with Diabetes Mellitus

Synthetic PPARγ agonists are used to improve insulin sensitivity in patients with diabetes mellitus; however, their use is limited by fluid retention [184,235,236,237,238]. This Na^+^ retention in nephrons may contribute to the development of edema and promote secondary hypertension in patients with type 2 diabetes mellitus, as a side effect of PPARγ treatment [62]. PPARγ agonists promote the activation of SGK1, the phosphorylation of Nedd4-2 and abolish ubiquitination and internalization of ENaC, leading to sodium and fluid retention [62]. Moreover, PPARγ stimulates Na^+^ transport in the distal tubular epithelia and proximal tubule cells via SGK1-dependent regulation of NHE3 [184,237,239]. Thus SGK1 contributes to the dysregulation of cellular Na^+^ and water transport in diabetes mellitus [184].

### 4.3. Cardiac Dysfunction

Dysregulation of Na^+^ homeostasis has been implicated in cardiac rhythm disorders as well as adverse ventricular remodeling [132,134]. SGK1 plays a pivotal role in early cardiac angiogenesis and vascular remodeling [135,240,241]. Chronic SGK1 activation in the heart increases mortality caused by cardiac arrhythmias [134,144]. This effect is paralleled by SGK1-dependent stimulation of the cardiac sodium channel Nav1.5 [134], the major influx channel responsible for the initiation of the cardiac action potential [132]. The SGK1-dependent upregulation of Nav1.5 alters sodium flux, leading to arrhythmia and cardiomyopathy [132,134,242].

Recent data suggested that the Na^+^/H^+^ exchanger NHE1, a target of SGK1, is involved in cardiac pathophysiology [143,144]. By increasing Na^+^ entry and subsequently decreasing the chemical Na^+^ gradient through a NHE1-mediated pathway, SGK1 contributes to myocardial remodeling, cardiac hypertrophy and progression to heart failure [78,134,143,144].

### 4.4. Implantation Failure

SGK1 has been detected in the human endometrium [243,244,245,246,247] and placenta [245,246,247,248]. Using a cDNA microarray, a previous study identified *SGK1* as a gene aberrantly expressed specifically in luminal epithelia during the midsecretory receptive phase of the cycle in infertile women [243]. In line with this, Salker et al. [246] confirmed that transcription of *SGK1* was higher in the uterine luminal epithelia of infertile women compared with fertile controls. They further demonstrated a transient down-regulation of *Sgk1* transcription in the mouse luminal epithelium during the window of endometrial receptivity [245,246,248]. Moreover, the expression of ENaC was upregulated, accompanied by the downregulation of Nedd4-2 in the *Sgk1^−/−^* mice [245,246,248]. In this respect, SGK1 expression and functional activation account for a successful implantation, by modulating ENaC activities and consequent fluid absorption before engraftment [248].

## 5. Conclusions and Perspectives

SGK1 is a prominent regulator of multiple Na^+^ channels, pumps and carriers, and thus contributes to the regulation of epithelial Na^+^ transport, cell volume and sodium homeostasis. This kinase is not expected to possess housekeeping functions, judging by the mild phenotype shown in both ubiquitous gene knockout and inducible tissue-specific *Sgk1* knockout mice [50,80,186,188,249]. By contrast, the gain of function of SGK1 is seemingly crucial for the pathophysiology of a wide variety of disorders [50,249]. Accordingly, SGK1 is thought to be involved in the formation of fibrosis which is characterized by dysregulated Na^+^ transport in several tissues [96,250]. Increased *SGK1* expression has been implicated in various fibrotic diseases, such as cystic fibrosis [96,251], renal fibrosis and albuminuria [250], diabetic nephropathy [250], glomerulonephritis [250], Crohn’s disease, fibrosing pancreatitis, and liver cirrhosis [48,50,249]. Additionally, as an osmosis-sensitive gene, *SGK1* might play a role in apoptosis, where cell shrinkage serves as a signal in programmed cell death or apoptosis [252]. In fact, downstream targets of SGK1, such as Na^+^/K^+^-ATPase and NHE1, are involved in cell apoptosis [252]. Recently, SGK1 has been proposed as a potential target of sodium intervention in immune cells [253,254]. NaCl affects the regulatory balance of type 1 helper T cell (T_H_1), T_H_2, T_H_17 and regulatory T cells (T_reg_ cells) in an SGK1-dependent manner [253,254]. In this regard, more studies are needed to determine whether SGK1 is a major driver or just a passenger in the pathophysiology of various disorders characterized by dysregulated sodium transport.

## Figures and Tables

**Table 1:** Serum and glucocorticoid regulated kinase 1 (*SGK1*) expressions throughout the body.

Organ	Distribution of *SGK1*	Reference
Brian	Hypothalamic nuclei	[16]
	Ventral striatum	[17]
	Dorsal horn	[18]
	Dopamine neurons	[19]
	Cortical pyramidal neurones	[20]
	Blood-brain barrier (BBB)	[21]
Eye	Ocular ciliary epithelium	[22]
	Corneal endothelium	[23]
Ear	Cochlear sensory epithelium	[24]
	Apical membrane of middle ear epithelium	[25]
Thymus	Epithelial cell	[2]
Heart	Heart chamber	[26]
Lung	Epithelial cell	[27]
Breast	Mammary gland	[28]
Liver	Epithelial cell	[29]
Pancreas	Pancreatic tissue	[30]
Intestine	Epithelium of the duodenum, jejunum, ileum, and colon	[31]
Kidney	Epithelium lining the nephrons (distal tubules, glomeruli, and inner medulla)	[32]
Bladder	Detrusor tissue	[19]
Muscle	Skeletal muscle	[33]
Adipose tissue	Adipocyte	[34,35]
Blood	Platelets	[36,37]
Immune system	T-lymphocytes	[38]
	Dendritic cell	[39]
	Macrophage	[40]
	Mast cell	[41]
Reproductive system	Several ovarian cell types including the oocytes of primordial follicles	[42]
	Sperm	[43]
	Primordial germ cell	[44]
	Decidua	[45]
	Placental trophoblast	[46]

**Table 2:** SGK1-dependent mediators of Na^+^ channels and transporters. *

Na^+^ Channels and Transporters	Mediators	SGK1 Regulation	Possible Mechanism	Reference
ENaC	Nedd4-2/14-3-3 protein	SGK1 phosphorylates and sequesters the negative regulator Nedd4-2. Meanwhile, SGK1 recruits 14-3-3 to stabilize Nedd4-2 interacting with 14-3-3	Nedd4-2 interacts with ENaC to induce ubiquitination and retrieval of ENaC channel; whereas 14-3-3 binds to Nedd4-2 and inhibits the interaction between Nedd4-2 and ENaC	[102,171]
	iNOS	SGK1 phosphorylates iNOS	NO reduces the open probability of ENaC	[172]
	AF9-Dot1a complex	SGK1 phosphorylates AF9 and promotes Dot1a to dissociate from the α-ENaC promoter	AF9-Dot1a complex facilitates Dot1a to hypermethylate Lys79 of histone H3 and suppress α-ENaC transcription	[126]
	WNK4	SGK1 phosphorylates WNK4	WNK4 inhibits ENaC activity	[118]
	NDRG2	SGK1 phosphorylates NDRG2 protein	NDRG2 stimulates ENaC activity and increase its surface expression	[33]
Nav 1.5	Nedd4-2	SGK1 phosphorylates and inactivates Nedd4-2	Nedd4-2 inhibits Nav1.5 activity	[135]
NHE1	14-3-3 protein	SGK1 recruits 14-3-3 binding	14-3-3 facilitates SGK1 to phosphorylate and stimulate NHE1	[143]
NHE3	NHERF2	SGK1 interacts with NHERF2	NHERF2 tethers SGK1 and NHE3 to facilitate the phosphorylation of NHE3	[141]
NCC	Nedd4-2	SGK1 Phosphorylates Nedd4-2 and abrogates Nedd4-2-mediated inhibition	Nedd4-2 is co-located with NCC and involved in the ubiquitylation of NCC transporter	[156]

* See text for abbreviations.
